# Unusual new signs of pneumothorax at lung ultrasound

**DOI:** 10.1186/2036-7902-5-10

**Published:** 2013-12-19

**Authors:** Giovanni Volpicelli, Enrico Boero, Valerio Stefanone, Enrico Storti

**Affiliations:** 1 Emergency Medicine, San Luigi Gonzaga University Hospital, Turin 10043, Italy; 2 Intensive Care Unit, Niguarda Ca’ Granda Hospital, Milan 20162, Italy

**Keywords:** Emergency ultrasound, Critical ultrasound, Lung ultrasound, Pneumothorax

## Abstract

**Background:**

The diagnosis of pneumothorax with a bedside lung ultrasound is a powerful methodology. The conventional lung ultrasound examination consists of a step-by-step procedure targeted towards the detection of four classic ultrasound signs, the lung sliding, the B lines, the lung point and the lung pulse. In most cases, a combination of these signs allows a safe diagnosis of pneumothorax. However, the widespread application of sonographic methodology in clinical practice has brought out unusual cases which raise new sonographic signs. The purpose of this article was to introduce some of these new signs that are described after the analysis of unusual and complex cases encountered during the clinical daily practice in an emergency department.

**Findings:**

The double lung point consists of the alternating patterns of sliding and non-sliding lung intermittently appearing at the two opposite sides of the scan. The septate pneumothorax allows B lines and lung pulse to be still visible in a condition of pneumothorax with absent sliding. In hydropneumothorax, the air/fluid border is imaged by lung ultrasound as the interposition between an anechoic space and a non-sliding A-pattern, a sign that may be named hydro-point.

**Conclusions:**

In bedside lung ultrasound, the operator should be aware and interpret double lung point, septate pneumothorax and hydro-point. The conventional diagnostic protocol of bedside lung ultrasound for pneumothorax should be occasionally adapted to such complex cases.

## Findings

### Introduction

Lung ultrasound is nowadays acknowledged as a useful methodology for the diagnosis of pneumothorax at bedside [1]. The diagnostic value of the sonographic signs of pneumothorax is similar to the well-known and largely used radiologic signs of the same condition in chest radiography. Visualization of a gap between the parietal and visceral pleura in a chest film is largely used, and intuitively diagnostic of pneumothorax, even though, to our knowledge there are no published studies specifically designed to evaluate its specificity in comparison with a gold standard. Nevertheless, there is not any doubt about the power and high specificity of this radiologic sign of pneumothorax. Similarly, the sonographic signs of pneumothorax are largely intuitive of the condition and share the same very high specificity with the radiologic signs [2,3]. Absence of lung sliding or pulse and absence of B lines combined with a lung point during a lung ultrasound examination have the same diagnostic meaning of the visualization of a pleural gap in a chest radiography. However, whereas chest radiography is a conventional method, lung ultrasound needed to break the wall of scientific prejudices before it was acknowledged as a viable method for a neumothorax diagnosis. This is the reason why many years and clinical studies were needed to change clinical guidelines and protocols and finally introduce bedside lung ultrasound in clinical practice. We cannot say anything unless this process has been completed. The need for a widespread use of lung ultrasound is compelling. If it is true, as it has been proven, that the specificity of a chest radiography is as high as a lung ultrasound, it is also true that many studies showed that sensitivity of lung ultrasound is far higher [1,4-6]. The category of radio-occult pneumothorax is part of the daily experience of any physician who faces trauma patients, post-procedural thoracic complications and spontaneous pneumothoraces.

### Sonographic diagnosis of pneumothorax

The bedside lung ultrasound diagnostic methodology for pneumothorax has been described in literature. A recent consensus process of the main experts in the field has led to the approval of a step-by-step methodology that consists of combining the four main sonographic signs of pneumothorax: the lung sliding, the lung pulse, the B lines and the lung point [1]. While the first three signs are strongly predictive of the absence of the condition, the lung point is the only sign that confirms a pneumothorax. However, the value of this general rule varies if we consider different settings and scenarios.

In the stable patient, the conventional methodology consists of scanning the most superior aspect of the chest with respect to gravity, bilaterally. In the supine patient, this area corresponds to the anterior-inferior chest. The probe should be applied on this area, on both sides. Detection of lung sliding and/or B lines is enough to conclude the examination and rule-out pneumothorax. Absence of sliding and B lines need to be confirmed by moving the probe towards the lateral chest to visualize the lung point. If the latter is detected, then the examination is concluded and the pneumothorax is diagnosed with an extremely high specificity. When the lung point cannot be detected, then absence of a lung pulse should also be checked. In this condition, absence of even the slightest pulsation of the subpleural lung image allows the diagnosis of a pneumothorax characterized by a complete pulmonary collapse. Conversely, even in the absence of sliding and B lines, visualization of a lung pulse rules out pneumothorax.

In the unstable patient or in a scenario of cardiac arrest, the methodology varies slightly. The most superior area of the chest is scanned for any sliding, pulse and B lines bilaterally. Absence of these three signs is enough to conclude the diagnostic process and move to an immediate life-saving invasive treatment. Faced with these extreme emergencies, there is no need to waste any more time checking for the presence of a lung point, because visualization of this latter sign would not change our decision to drain. Indeed, if we do not detect a lung point, then a tension pneumothorax is the diagnosis and we should treat it in the immediate; if we visualize a lung point in any part of the chest, the pneumothorax needs to be treated anyway. Instead, visualization of even one among sliding, pulse or B lines allows ruling out pneumothorax as the cause of the clinical storm.

The procedure described so far is generally accepted and widely applied. However, there are some exceptions that should be always considered and known by the operators. These conditions represent sonographic challenges and may be indicated in the category of *complex pneumothorax*.

### Sonographic signs of complex pneumothorax

**Figure 1:**
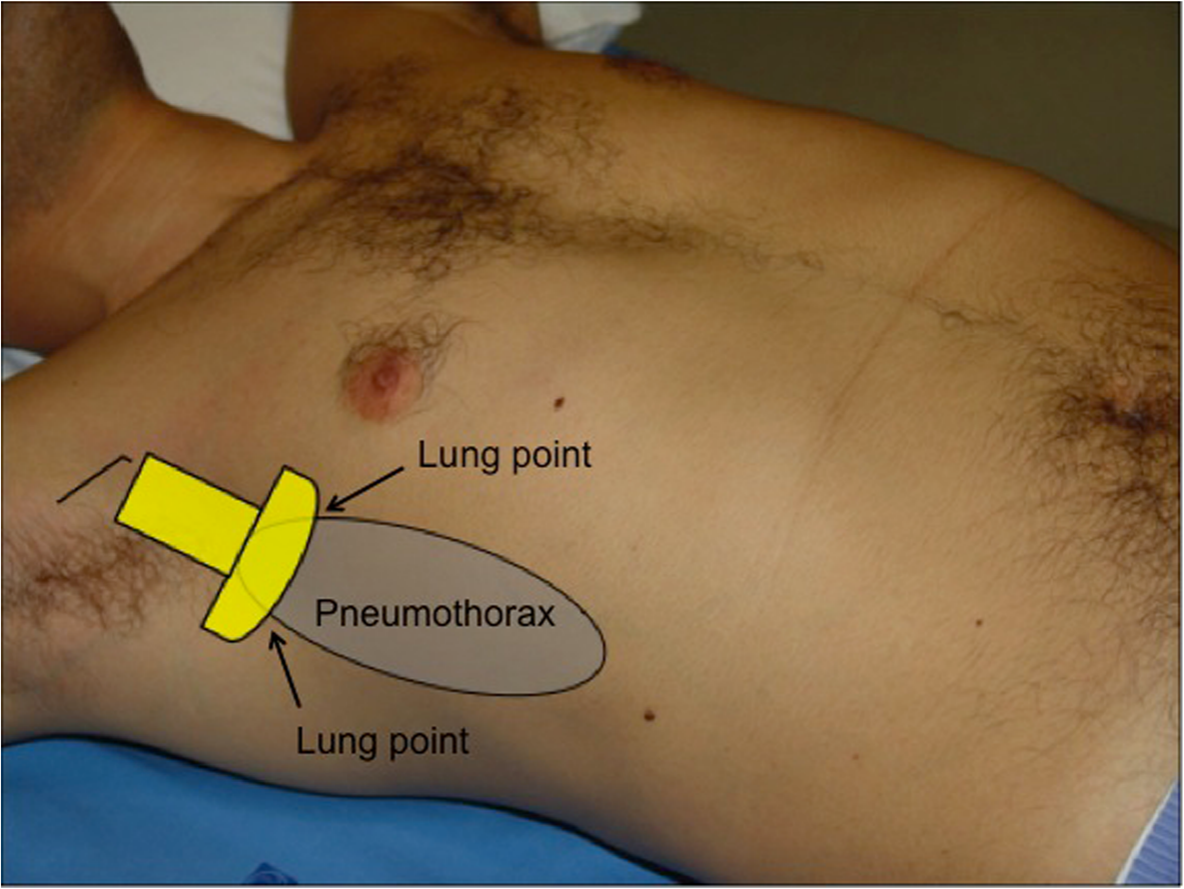
Visualization of the two edges of the air trapped in the pleural space.

*Double lung point*: when for some reason the air of a pneumothorax is not free to float inside the pleural space, a minimal amount of pleural air may remain in the lateral or dorsal chest without migrating in the most superior area in a supine patient, which corresponds to the anterior-inferior chest zone. In this case, the operator may visualize two lung points, i.e. the alternating patterns of sliding and non-sliding lung intermittently appearing at the two opposite sides of the scan (Additional file 1) [7,8]. These two lung points represent the visualization of the two edges of the air trapped in the pleural space (Figure 1). Pneumothorax with air trapping may be caused not only by pleural adherences in chronic pleural and pulmonary diseases but also by acute lung contusions in blunt torso trauma [9]. Even without abnormal pleural adherences, very small spontaneous pneumothoraces may not have enough pressure to allow complete detachment of the pleural layers and the floating of air towards the most superior chest areas [7]. Being aware of this condition or in case of strong suspicion, the operator should always complete the scan of the lateral chest in the supine patient to confirm lung siding even when this latter is first visualized in the parasternal anterior-inferior chest. In the unstable patient, this extension of the technique is less important. Presence of lung sliding in the anterior-inferior chest may conclude the ultrasound examination, unless the patient is intubated for pressure ventilation or is going to be transported by helicopter [10]. In these two latter cases, the lateral chest should always be scanned to rule out even the smallest pneumothorax that may need to be monitored or warrant prophylactic drainage.

**Figure 2:**
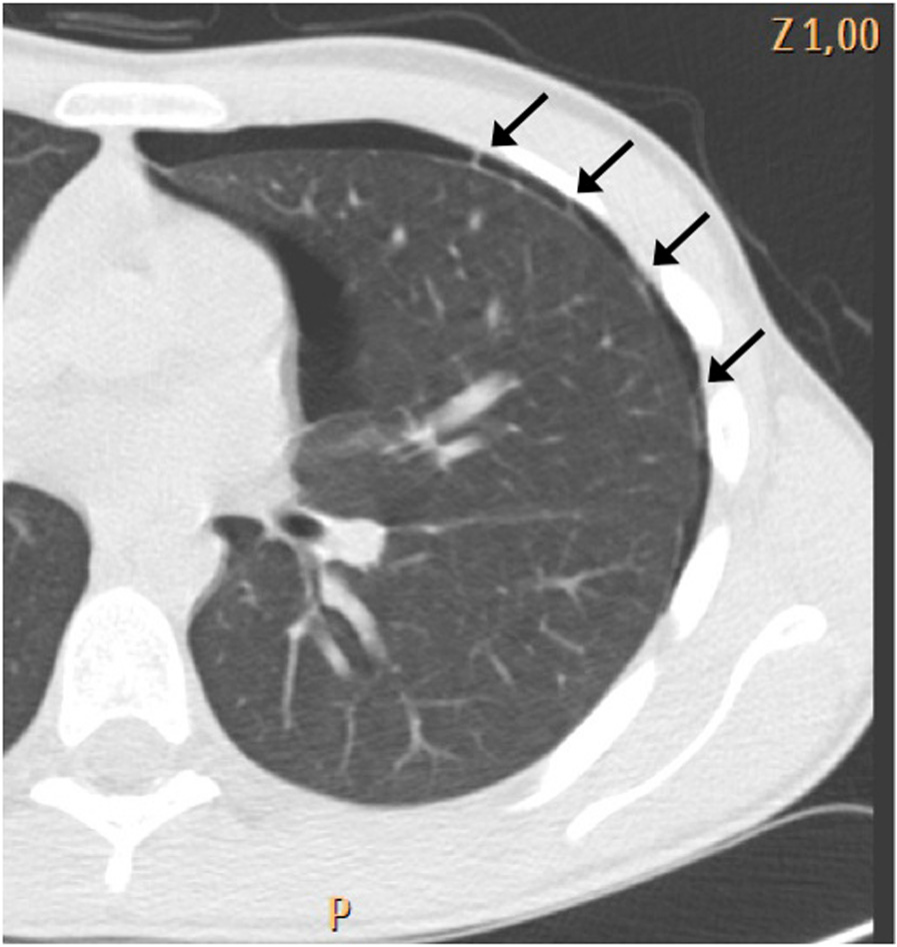
The small areas showing B lines and lung pulse correspond to small pleural adherences.

*Septate pneumothorax*: recurrent pneumothoraces after invasive therapeutic procedures are often characterized by abnormal ultrasound findings. In patients with failed pleurodesis, it is quite common to observe the typical ultrasound pattern of septate pneumothorax [11]. In this case, the absence of sliding may be combined with the persistence of B lines and lung pulse in the same scan (Additional file 2). While, in the majority of patients, visualization of B lines and lung pulse rules out pneumothorax, there are rare cases where the negative predictive power of B lines and lung pulse may be misleading. In the context of absent lung sliding, the small areas showing B lines and lung pulse correspond to small lung regions where the parietal and visceral pleura are still touching due to the presence of septa (Figure 2). Demonstration of a lung point in other areas of the chest is a decisive step to conclude the examination and diagnose pneumothorax. A sonographic pattern that combines an absence of lung sliding but presence of B lines and/or lung pulse with presence of a lung point is diagnostic of septate pneumothorax.

**Figure 3:**
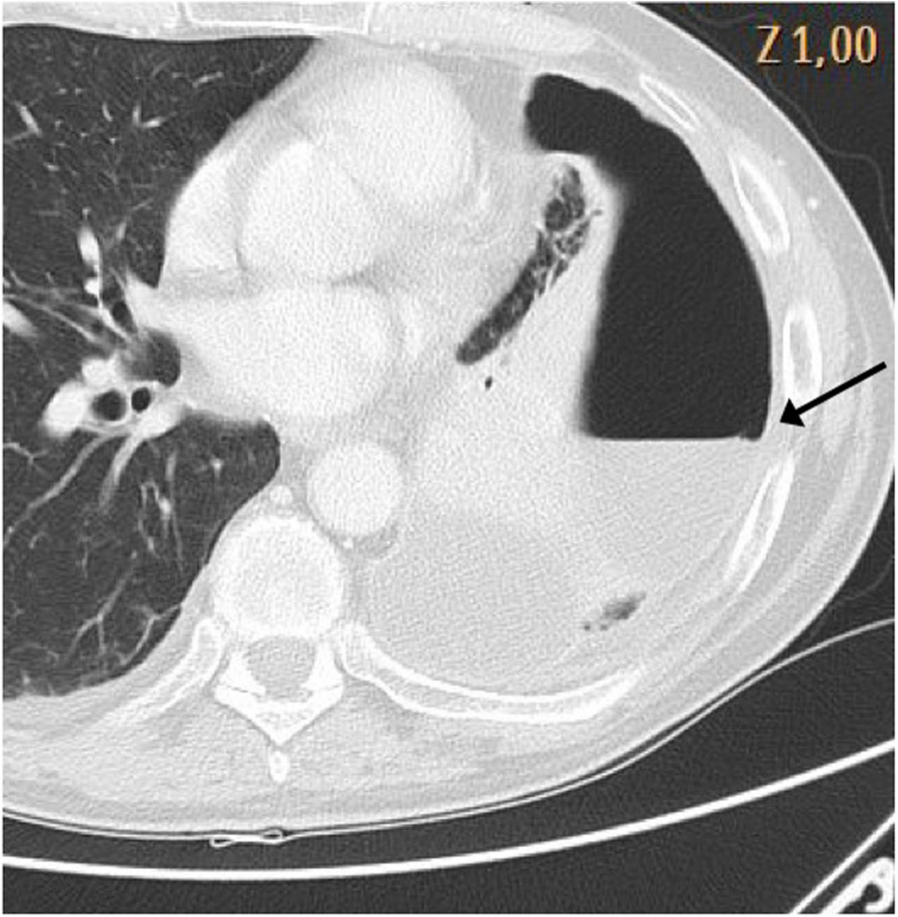
Opposition between the air/fluid patterns is the hydro-point.

*Hydropneumothorax*: iatrogenic pneumothorax following procedures of thoracentesis in pleural effusion is a well known complication. While interposition between the normally aerated lung and pneumothorax (air/air interface) is demonstrated in a lung ultrasound by the lung point sign, air/fluid interface in the pleural space gives a different sonographic pattern. In hydropneumothorax, the pleural effusion is demonstrated by the visualization of space, usually anechoic, between the two pleural layers while pneumothorax gives the well-known A pattern, i.e. the reverberation of the chest wall image below the pleural line with A lines, absence of sliding or pulse and absence of B lines (Additional file 3). Opposition between these two patterns is the hydro-point (Figure 3). This recently described sonographic sign shares the same diagnostic power with the lung point for the diagnosis of pneumothorax [12].

### Conclusion

Lung ultrasound is rapidly spreading as a safe bedside methodology for the diagnosis of pneumothorax in different settings. Because of its increasing use in the clinical practice, observations of some unusual and complicated cases are also emerging. The conventional step-by-step sonographic technique and the four conventional ultrasound signs of pneumothorax should be slightly modified to consider the possibility of facing complex cases. Complicated pneumothorax may be encountered in many different settings, such as trauma patients, spontaneous pneumothorax, recurrent pneumothorax after pleurodesis and post-procedural pneumothorax. The operator should be aware and know how to interpret unusual sonographic signs and patterns, such as the double lung point, the septate pneumothorax and the hydro-point.

## Competing interests

The authors declare that they have no competing interests.

## Authors’ contributions

GV observed the cases and collected the videos, conceived and wrote the manuscript. EB, VS and ES contributed to collect the videos and to write the manuscript. All authors read and approved the final manuscript.

## Supplementary Material

Additional file 1 ***The double lung point*: video shows the alternating patterns of sliding and non-sliding lung intermittently appearing at the two opposite sides of the scan.** This pattern represents the visualization of the two edges of the air trapped in the pleural space. Click here for file

Additional file 2 ***Septate pneumothorax*: video shows presence of B lines and lung pulse in a condition of absence of lung sliding.** The areas where B lines and pulse are still visible correspond to small lung regions where the parietal and visceral pleura are still touching due to the presence of septa. Click here for file

Additional file 3 ***Hydropneumothorax*: video shows the interposition between a fluid filled anechoic space and the lung non-sliding A-pattern.** This sign is the hydro-point and has the same meaning of the lung point. Click here for file
